# An integrated approach to meet the needs of high-vulnerable families: a qualitative study on integrated care from a professional perspective

**DOI:** 10.1186/s13034-020-00321-x

**Published:** 2020-05-09

**Authors:** L. A. Nooteboom, S. I. van den Driesschen, C. H. Z. Kuiper, R. R. J. M. Vermeiren, E. A. Mulder

**Affiliations:** 1 Department of Child and Adolescent Psychiatry, Curium-Leiden University Medical Centre, Post Box 15, 2300 AA Leiden, The Netherlands; 2 Leiden University of Applied Sciences, Zernikedreef 11, 2311 CK Leiden, The Netherlands; 3 Horizon Youth Care and Special Education, Mozartlaan 150, 3055 KM Rotterdam, The Netherlands; 4 Youz: Parnassia Group, Dr. van Welylaan 2, 2566 ER, The Hague, The Netherlands; 5 Intermetzo-Pluryn, Post Box 53, 6500 AB Nijmegen, The Netherlands; 6 Department of Child and Adolescent Psychiatry, Amsterdam University Medical Centre - location VUMC, Meibergdreef 5, 1105 AZ Amsterdam, The Netherlands

**Keywords:** Integrated Care, Child and Adolescent mental health, Professionals, Care provision, Families

## Abstract

**Background:**

To meet the needs of high-vulnerable families with severe and enduring problems across several life domains, professionals must improve their ability to provide integrated care timely and adequately. The aim of this study was to identify facilitators and barriers professionals encounter when providing integrated care.

**Methods:**

Experiences and perspectives of 24 professionals from integrated care teams in the Netherlands were gathered by conducting semi-structured interviews. A theory-driven framework method was applied to systematically code the transcripts both deductively and inductively.

**Results:**

There was a consensus among professionals regarding facilitators and barriers influencing their daily practice, leading to an in depth, thematic report of what facilitates and hinders integrated care. Themes covering the facilitators and barriers were related to early identification and broad assessment, multidisciplinary expertise, continuous pathways, care provision, autonomy of professionals, and evaluation of care processes.

**Conclusions:**

Professionals emphasized the need for flexible support across several life domains to meet the needs of high-vulnerable families. Also, there should be a balance between the use of guidelines and a professional’s autonomy to tailor support to families’ needs. Other recommendations include the need to improve professionals’ ability in timely stepping up to more intensive care and scaling down to less restrictive support, and to further our insight in risk factors and needs of these families.

## Background

It is a major challenge for professionals in Youth Care to timely and adequately meet the needs of high-vulnerable families [1]. Although a small group (e.g. 3–5% of all families in the Netherlands; [2]), these families are in need of support from multiple services due to severe and enduring, co-occurring problems across several life domains (e.g. mental health, parenting, financial or housing, somatic health, criminal activities, substance abuse; [3]). While providing integrated support has been recognized as a necessity [4], the support of high-vulnerable families is often complicated by the chronic, unpredictable nature of co-occurring and interacting problems in multiple family members (both child and parental factors), and by families’ reoccurring crisis situations [3]. If left unsupported due to a lack of treatment, interventions, or assistance, these problems and situations cause distress and impairment with life-long consequences on psychosocial functioning in children, their families, and the community [5]. Furthermore, feeling unable to support these families can lead to work-related stress, poor well-being, and an increased risk of burnout in professionals [6].

Currently, support for high-vulnerable families in Youth Care is performed by multiple professionals from different organizations, for example professionals from community centres, (special) education, specialized mental health care, child protection, parenting support, social work, and residential treatment. Youth Care is defined as the support for children aged 0–25 and their families including a wide range of services: from universal and preventive services to specialized care [7]. Previous studies stressed that interprofessional collaboration is at present, however all too often characterized by fragmentation of (costly) services, resulting in a lack of coherence and coordination in the care process [8, 9]. Subsequently, high-vulnerable families can present resistance to the support from Youth Care professionals. It is unclear whether these families actively resist support due to their negative experiences with prior support or difficulties in forming therapeutic alliance [10], or whether they do not receive the support they need. To overcome these difficulties, there is a need to substantially improve professionals’ ability to support these families in an integrated way.

In integrated care, professionals aim to collaboratively address a wide variety of problems at different levels and sites within the continuum of care in a coordinated, coherent, and continuous way [4]. As reported in previous research [8, 11, 12], a necessity to meet the needs of families is to align available support throughout the entire continuum of care (e.g. from primary care to highly specialized mental health care). According to leading approaches, integrated care provision can be simultaneous, with varying intensity tailored to families’ needs (matched care), or sequential by increased intensity of support (stepped care). In matched care, families are allocated (‘matched’) to support based on the assessment of individual needs, risk factors, characteristics, and values [13, 14]. Since support is tailored to individual needs, it varies across clients regarding intensity, setting, and type of services [13]. The alternative approach, stepped care, is about offering the least restrictive support that is still likely to yield significant health gain, and ‘step up’ to more intensive support if needed by a predefined evidence-based sequence of options for support [15–17]. Stepped care is self-correcting, meaning that progress and response to support are reflexively monitored and systematically evaluated by professionals and clients to assess if support must be altered [16, 18, 19]. For clients with single problems, stepped care was found to be effective in terms of clinical outcomes, cost-effective allocation of resources, and efficiency of support [13, 15, 18, 20].

Theoretically, matched and stepped care seem distinct. However, in clinical practice these approaches are difficult to distinguish and often applied interchangeably in an unthoughtful way. Moreover, in both matched and stepped care there is a lack of predefined criteria and guidelines for monitoring, evaluating, and applying the most appropriate and available support based on families’ multiple needs [13]. Furthermore, guidelines rarely consider decision making for families with multiple interacting problems and do not take social circumstances or individual preferences into account [21]. This can lead to intuitive decision making by professionals and inadmissible variations in support due to different values, perspectives, and expertise of professionals [13, 16]. The interaction and unpredictable nature of the broad variety of co-occurring problems complicates the matching of individual family members to the most suitable and available support [13]. As a result, some families may receive excessive support, while others are insufficiently supported, leading to inappropriate care provision and inefficient allocation of resources [22]. Furthermore, a difficulty with sequencing in stepped care reported in previous studies [23–25] is the individual and disease-specific focus, overlooking the interaction of problems and leading to fragmented support offered by multiple professionals and organizations. Another difficulty in stepped care is that failure of the least restrictive support can negatively affect families’ motivation, eventually leading to resistance of families to support and high risks of drop out [26].

Altogether, these difficulties often result in inappropriate, delayed, or prolonged trajectories, or no care provision at all. Consequently, problems exacerbate, leading to further impairment in functioning of high-vulnerable families [27], increased costs, and burden on the relatively scarce professionals and services such as specialized mental health care [28, 29]. In addition to governmental policy concerns and changes at organizational level by forming networks and aligning services, there is a need to substantially improve professionals’ ability to support these families in an integrated way [1, 30]. Therefore, this qualitative study aims to identify facilitators and barriers professionals encounter when providing timely and adequate integrated care to these families. Actual experiences and perspectives of professionals in the field of Youth Care that work in integrated care teams will be translated into insights and recommendations for professionals, their organizations, researchers, and governmental policy makers.

## Methods

### Setting

This study is part of a larger research project which focusses on integrated care teams for children and their families in the Netherlands. In the Netherlands, municipalities are responsible and have the authority to organize Youth Care on a local level, including preventive services, youth mental health care services, and specialized Youth Care [7]. The presumed improvement of organizing Youth Care on a local level is that integrated care can be provided at an earlier stage, within the family’s own environment, and with easy access to various local services. In almost all municipalities, so called Youth Teams operate within a primary care setting, as a linking pin between preventive services and specialized mental health care [7]. Youth Teams are multidisciplinary teams consisting of eight to twelve professionals with different expertise (i.e. social work and education, specialized mental health care, infant mental health care, support for youth with (mild) mental retardation, coaching, parenting support, and child protection). Youth Team professionals can coordinate a care process and provide short-term support if needed. They operate following both matched and stepped care approaches: professionals tailor support based on families’ needs and characteristics (‘matched care’), and if needed, they refer to appropriate support in steps of increased intensity (‘stepped care’), starting with the least restrictive as possible.

### Participants

Professionals were invited to participate in semi-structured interviews by one of the researchers (LN) during their weekly team meetings. To obtain a representative and complete sample of Youth Team professionals, we aimed to include at least three professionals from each of the six participating Youth Teams. There were no further inclusion or exclusion criteria, since we intended to target a heterogeneous group of Youth Team professionals with diverse expertise (e.g. (infant) mental health, social work and education, (mild) mental retardation, child protection, and parenting support). Convenience sampling was applied based on availability since all professionals were capable of providing adequate information about their experiences in integrated Youth Teams [31]. None of the participants refused to participate after application for the interview. There was some degree of acquaintance between participants and the researcher because of their participation in the overall research project. However, the students who conducted the interviews under supervision had no prior knowledge of the participants. Interviews were scheduled at the professionals’ work place in a separate room. Participants were verbally informed of the study aims and interview procedures, and subsequently provided written informed consent. Participants were asked to fill in a demographic questionnaire after each interview.

The Medical Ethics Review Board of Leiden University Medical Centre judged that the overall research project should not be subject to evaluation based on the Medical Research Involving Human Subject Act (WMO) and complied with the Netherlands Code of Conduct for Research Integrity. Reporting of the study methods and results was informed by the Consolidated Criteria for Reporting Qualitative Research (COREQ; 32).

### Data collection

Semi-structured interviews were conducted between July and August 2017 by a student of the University of Applied Sciences in Leiden (DN, male or ET, female) under supervision of a trained interviewer (LN or JE, both female). The interviews were guided by a topic list with open-ended questions to facilitate deep understanding of viewpoints and experiences of professionals [33]. The topic list was formulated in advance based on previous reviews on integrated care [1, 8], and supplemented by input from reflexive meetings of the researchers. Subsequently, the topic list was pilot tested on four professionals from different Youth Teams who were involved in the overall research project. The topics focused on: the general working method of professionals, a professional’s expertise to support a broad range of problems in Youth Care, early assessment and identification of problems, clinical decision making, interprofessional collaboration within the Youth Team, interprofessional collaboration with other stakeholders, availability of support, and timely step up or scale down to appropriate support. All interviews were conducted in Dutch, audio-recorded, and transcribed verbatim to avoid interpretation bias [34]. Field notes were obtained during the interviews. No participant expressed interest in commenting on the Dutch transcripts. The presented quotes in the result section were translated literally from Dutch to English by two researchers (LN, SvdD). Hence, the quotes contain literal wordings and might not be completely fluent.

### Analysis

All transcripts were imported into the computer program ATLAS.ti (version 7) for coding and analyzing the text content. A framework method was applied to systematically code the transcripts by following a standardized procedure to maintain a transparent audit trail and enhance the rigor of the analytical process [35, 36]. The coding framework (Appendix 1: Table 2) was built by combined qualitative analysis, both deductively and inductively [36]. First, codes were deductively formulated based on previous literature on integrated, stepped, and matched care (LN, SvdD, CK). Facilitators were conceptualized as components enabling professionals to provide integrated care. In contrast, barriers were defined as components limiting integrated care in practice. After familiarization with the transcripts, the framework was pilot-tested on two interviews by two researchers independently (LN, SvdD). After resolving uncertainties and differences, the framework was applied on all the interviews by the two researchers. During the coding process, the framework was supplemented with codes generated from inductive, open coding. After five interviews, no new codes were formulated, an indication that we built a comprehensive coding frame. We applied this coding framework on all the following interviews to identify the barriers and facilitators. Subsequently, axial coding took place by further analysis and merger of the coded fragments, resulting in themes that covered the broad variety of facilitators and barriers. The data was interpreted back and forth as an iterative process [35], supplemented by reflexive meetings (LN, SvdD) in between each interview to discuss the coding and interpretation process. By applying this bracketing method we aimed to limit possible adverse effects of prejudices [34]. Inductive thematic saturation was reached after analyzing 17 interviews [37].

## Results

### Demographics

In total, 24 professionals (2 male and 22 female) participated in the interviews, 4 from each Youth Team. This male–female ratio reflects the actual gender representation in Youth Teams in the Netherlands. The interview duration ranged from 39 min to 79 min (M = 56 min). Participants’ education varied and they held various areas of expertise (e.g. social work and education, specialized mental health care, infant mental health, (mild) mental retardation, coaching, parenting support, and child protection). See Table 1 for an overview of the demographic characteristics of the professionals.

**Table 1:** Demographic characteristics of the professionals

Variable	
Gender	
Male [n (%)]	2 (8.3%)
Female [n (%)]	22 (91.7%)
Age in years	
Mean age in years (SD)	39.25 (11.04)
Age range in years	24–61
Highest educational level	
Higher vocational education [n (%)]	21 (87.5%)
University [n (%)]	3 (12.5%)
Area of expertise	
Socio-pedagogical assistance [n (%)]	11 (45.8%)
Pedagogics [n (%)]	6 (25.0%)
Psychology [n (%)]	1 (4.2%)
Social work [n (%)]	5 (20.8%)
Music therapy [n (%)]	1 (4.2%)
Years of work experience	
Mean years of experience (SD)	14.23 (9.67)
Range years of experience	1.5–35

N = 24

### Findings

Overall, there was a consensus among professionals regarding the reported facilitators and barriers that influenced the provision of integrated care. As a result, the interviews were largely complementary. Based on the thematic analysis of the reported barriers and facilitators, six themes were formulated:

1. Early identification and broad assessment to timely recognize potential risk factors.
2. Multidisciplinary expertise: specialist professionals in a generalist team.
3. Continuous pathways: flexible support throughout the entire continuum of care.
4. Current approaches in integrated care provision: a mix of stepped and matched care.
5. Autonomy of professionals: tailor support and follow guidelines.
6. Evaluation of care processes: discuss progress and alter support if needed.

Results are presented in the following section, starting with general aspects of integrated care and followed by a thematic report of the facilitators and barriers. An overview of facilitators and barriers per theme can be found in Appendix 2: Table 3, the frequency of quotes per code can be found in Appendix 1: Table 2.

### General aspects of integrated care

Most professionals found it difficult to define integrated care. In general, descriptions were related to interprofessional collaboration. Professionals mentioned for example colocation, the presence of a Youth Team professional at schools or other sites in the neighborhood. Professionals also described integrated care as a central access point for multiple services; working towards mutual goals; coordination; and sharing responsibilities. On the other hand, some professionals referred to integrated care as a holistic, family-centered approach, focusing on the needs of all family members across multiple life domains. These professionals emphasized that a family-centered approach is crucial in integrated care, since the problems of one family member often impact the entire family’s functioning. To provide integrated care, the aim of most professionals was to look beyond the initial request for support and broadly assess the entire family’s functioning.

*“Integrated is of course a very broad concept. That you obtain knowledge on several areas of life: the family level and how they are related to their context, the environment and those involved. In that way, I understand integrated care for families. That you obtain knowledge of their functioning and that you provide support on those aspects if needed.”*

– Professional HR3.3 -

Professionals found it challenging to support high-vulnerable families. Most professionals described the combination of (mild) intellectual disability, psychiatric problems, and safety concerns as demanding in view of the chronicity, interaction, and unpredictability of these problems. Collaboration between Youth Team professionals and services focusing on adults was considered a necessity to coherently support the entire family. However, this collaboration was often complicated by fragmentation between youth- and adult services. Another barrier to a family-centered, integrated approach was the resistance of parents the moment professionals attempted to discuss parental problems, particularly when the initial request for support focused on the child’s malfunctioning.

### Theme 1: Early identification and broad assessment to timely recognize potential risk factors

The first theme was timely recognition of (potential) risk and protective factors across several life domains by early identification and broad assessment of problems. To adequately support high-vulnerable families, most professionals did not feel that they had to *solve* all problems a family encountered, but that their task was to *identify* families’ needs and timely involve other professionals with the required expertise if needed. Reported facilitators to early identification of potential vulnerable families were early consultation; being aware of potential risk factors and intergenerational transmission of problems; enhanced accessibility of support by offering free trainings; and one visible point of entry for families. Early consultation was often established by professionals’ colocation at schools, general practitioners practices, police centers, or at youth health care centers. This requires availability of professionals, an outreaching approach, and familiarity with other systems and their work-flow. A reported barrier to early identification was the risk of providing excessive support to families with minor problems. To prevent professionals from doing so, adequate triaging is needed.

*“By adequately identifying signals and from there, I assess what is needed. I also think that [professionals should possess] general knowledge of the possibilities and which intervention suits best. And then I can see if it is something that I can do myself, or if it is something that I have to refer to specialized mental health care services.”*

- Professional HR1.3 -

Professionals stressed that broad assessment at the beginning of a care process is essential to identify needs across several life domains. Reported facilitators were addressing a broad range of topics and the use of a shared care plan. Professionals described the following topics for broad assessment: complaints and strengths; functioning across several life domains (at home, at school/work, in the community); involvement of previous/current professionals and services; and the informal (social) network of families. Furthermore, formulating a care plan in collaboration with families facilitated an overview of families’ functioning across several life domains.

On the other hand, some professionals reported barriers to broad assessment, including a lack of knowledge on a broad range of problems and the burden broad assessment might put on families. Although most professionals felt confident and competent to make an initial assessment of a family’s needs, one professional stressed that a lack of knowledge was a barrier to ask about problems that felt outside her field of expertise. Furthermore, broad assessment was often considered as time consuming and burdensome for families, since families had to share detailed personal information at the beginning of a care process while the relationship with their professional was not yet established.

### Theme 2: Multidisciplinary expertise: specialist professionals in a generalist team

“*It is not that I am an expert in all areas of expertise. But I have general knowledge of most areas of expertise as a generalist, and I have specialists in my team who know the rest*.”

- Professional DH2.1 -

Regarding multidisciplinary expertise, the second theme we identified, professionals emphasized the need of both generalist and specialist expertise to provide integrated care. In that, professionals stressed the importance of being aware of the reach of their own expertise. Specifically, professionals described the importance of recognizing the boundaries of their expertise and timely involving professionals with other expertise if needed. The multidisciplinary character of Youth Teams was described as a facilitator to integrated care since the multidisciplinary teams deployed a broad range of expertise in one place to support families with multiple needs; professionals were able to take different roles towards families during a care process; and it enabled them to learn from another professionals’ expertise. To facilitate interprofessional collaboration within a Youth Team, professionals often worked in pairs and held weekly multidisciplinary case discussions with the entire team. To avoid a multidisciplinary team full of generalists, professionals stressed the importance of keeping their expertise up to date. Professionals thought it was the responsibility of organizations to accommodate specialist training and supervision. A reported barrier was the high working demand, forcing professionals to provide support on areas outside their own expertise. This did not only decrease the quality of support for families, but also felt unsafe for professionals.

### Theme 3: Continuous pathways: flexible support throughout the entire continuum of care

The third theme, continuous pathways, can be described as clear, coherent, and coordinated alignment of support throughout the entire continuum of care. According to most professionals, high-vulnerable families need a flexible provision of support through the continuum of care with varying intensity, that is matched to a family’s changing needs. Professionals described various facilitators for continuous pathways:

- *Familiarity* with other professionals and their working approaches, leading to increased trust and improved interprofessional collaboration. Co-location and joint case discussions were reported facilitators to increasing familiarity.
- *Frequent evaluation and long*-*lasting agreements* with all professionals involved in care processes throughout the entire continuum of care.
- *Sharing up to date information* with other professionals, based on mutual agreements on the content and frequency of sharing information.
- *Warm handoff*, described as the gradual transfer from one professional or organization to another.
- *A care coordinator*, described as a professional who maintains an overview of the care process. The care coordinator facilitates communication between professionals involved, and coordinates support in line with families’ needs. Whether this care coordinator can also provide ambulatory support to a family remained unclear from the interviews, since professional perspectives varied at this point.

“*That families are being monitored, or no, receive continuous support. The moment it improves, professionals can take a little more distance, and if needed, they can return to support the family.”*

- Professional DH2.2 -

On the other hand, professionals described multiple barriers for continuous pathways. First, coherent and continuous support was often hampered by the complexity and variability of families’ problems. In supporting high-vulnerable families, the responsibilities, tasks and roles of the professionals involved were often unclear, leading to fragmented support and confusion by both families and professionals. Other reported barriers were the high turnover rates of professionals, the time-consuming process of interprofessional collaboration, and specific organizational demands, for example requiring professionals to stay involved in a care process as short as possible. Professionals’ unavailability hindered warm handoffs, just as privacy issues were reported as a barrier to sharing information.

Another barrier to form continuous pathways, reported by all professionals, was the lack of availability of support often due to long waiting lists. This led to a delay in care provision, sometimes for over half a year. Consequently, professionals who were already involved in the care process felt responsible or forced to provide inadequate support during these transition times. Besides the risk of increased complaints and drop out of families, this inadequate support also burdens professionals and reduces the quality of support. Alongside the long waiting lists, availability of support also seemed limited for specific ethnic groups such as immigrants and non-native speakers. Professionals described the limited ethnical diversity of professionals employed in Youth Teams and language barriers as reasons for this specific lack of availability.

### Theme 4: Current approaches in integrated care provision: a mix of stepped and matched care

This fourth theme is about current approaches in integrated care provision: stepped and matched care. Based on the interviews we conclude that professionals offered a mix of matched and stepped care in practice. Professionals reported starting with the least restrictive support as possible and gradually increase intensity of support if needed. On the other hand, professionals described that they tailor support to families’ needs and immediately referred families to more intensive support if necessary. In the following section, the application of matched and stepped care in practice is discussed, followed by facilitators and barriers to timely stepping up to more intensive support and scaling down to less restrictive support.

### Matched care

Matched care was described as tailoring support to families’ needs and preferences based on their demands. Matched care was explained as the opposite of a supply-oriented approach which involves allocating support based on services, offered by organizations. Professionals intended not only to tailor support based on the severity of problems, but also on families’ preferences regarding the location, type of service, and frequency of visits. In that, professionals stressed that families were not completely free in their choices and emphasized the need for shared decision making. Reported facilitators to shared decision making were the provision of different options for support, and taking both the professional’s appraisal and families’ preferences into account. Professionals emphasized the need to guide parents through the decision-making process by adjusting their pace, offering multiple choices, considering different preferences between family members, and considering cultural differences.

*“Sometimes the mother asks for a psychologist. Yes… but mother can ask all she wants, we do not always offer everything a parent wants. Maybe it is more a general request for help, a cry for a psychologist while all mother really wants is being heard. And when you can ask as much as possible beyond this initial request, the faster you can provide adequate support.”*

- Professional DH3.2 -

### Stepped care

In general, three aspects of stepped care were described by professionals: starting with the least restrictive option for support by involving the social network or volunteers; allocating support by an increased intensity, from preventive to more intensive support; and following a predetermined sequence of steps.

“*Working by a stepped care approach can also just be that you start with groups, and afterwards start an individual trajectory. In this way, you may also ensure a reduction in waiting lists. Because you see people in groups, you can offer support quicker and eventually, perhaps 40% of the people on a waiting list are sufficiently supported by a group training.*”

- Professional HR1.3 -

According to some professionals, a stepped care approach ensured more effective evaluation of a family’s goals and provided structure during a care process. Overall, professionals reported two major barriers to applying a stepped care approach. First, although starting with the least restrictive form of support was sufficient for some families, for high-vulnerable families this was often inappropriate, increasing the risk of providing insufficient support, drop out, and dissatisfaction. Second, there was often a time-limit for each step based on a protocol that did not match the pace of families (e.g. the number and length of visits). As a result, support was not tailored to families’ needs.

### Stepping up and scaling down

Both in matched and stepped care, stepping up to more intensive support and scaling down to less restrictive support were reported as important elements to ensure adequate allocation of support. Multiple professionals described that specific expertise was needed to step up and scale down adequately in collaboration with families. In both stepping up and scaling down, professionals stressed the following facilitators: a future-oriented care plan formulated in collaboration with parents; early involvement of the informal (social) network and schools; and frequent evaluation of a family’s progress.

“*I am very much in favor of preventive services to stimulate parents in solving their problems independently and voluntarily. But sometimes that is simply not possible. And if things remain within voluntary support for too long before referring to more intensive, restrictive support… Then so much has been tried and there is so much resistance, that in the restrictive setting things are difficult to change, because parents simply do not want anymore.”*

- Professional DH2.1 -

In stepping up, professionals were hindered by difficulties in early assessment, a lack of availability of support, and resistance of families. Stepping up too late negatively influenced care processes and resulted, due to exacerbation of problems, in prolonged care processes and a crisis-oriented focus of support. Professionals experienced multiple barriers to scaling down. First, limited attention was paid to scaling down and timely introducing less restrictive support to families during care processes. As a result, intensive support trajectories ended too abruptly or continued for too long. Second, in supporting high-vulnerable families who are hallmarked by their instability and high risk of relapse, professionals encountered difficulties in objectively assessing families’ actual needs, leading to scaling down too late. Other reasons for a delay in scaling down were the experienced sense of responsibility, professionals’ personal involvement, and the resistance of families towards less restrictive support, for example provided by volunteers.

### Theme 5: Autonomy of professionals: tailor support and follow guidelines

The fifth theme was autonomy of professionals: the freedom professionals experienced in their daily practice. Professionals described the autonomy to undertake a variety of tasks and tailor support to a family’s needs as a facilitator to integrated care. Professionals reported valuing their autonomy since it led to an increased focus on a professional’s competencies and room for personal development. On the other hand, autonomy was reported as a barrier. Some professionals experienced too much autonomy in their work due to unclear tasks and vague responsibilities, leading to feelings of insecurity. Also, professionals stressed that too much autonomy could lead to inadmissible differences in the type of support families with similar problems receive. To reduce this disparity, professionals stressed the importance of discussing the focus of support within their multidisciplinary Youth Team.

*“It is also a bit overwhelming, because as a professional you need boundaries so you know how to handle certain situations; what works in a specific situation, based on scientific research. It similarly gives much freedom, although such freedom can be a bit overwhelming.”*

- Professional DH3.4 -

Professionals reported that they applied a selection of elements from guidelines or protocols in their daily practice based on their own assessment. Many professionals reported that following fixed protocols or evidence-based guidelines was limiting their autonomy. On the other hand, there were professionals who stressed that guidelines offered structure, extended their expertise, and resulted in more aligned care processes. A small group of professionals mentioned the limited use of guidelines as controversial, since it increases the risk of intuitive decision making, varying working approaches, and might decrease the effectiveness and quality of support.

### Theme 6: Evaluation of care processes: discuss progress and alter support if needed

The sixth and last theme we formulated was evaluation: keeping track of a care process by monitoring and discussing the progress and timely altering support if needed. Professionals described evaluation on three levels: evaluation of the care process together with families; multidisciplinary case discussions within a Youth Team; and evaluation of collaboration with professionals of other organizations. For all levels of evaluation, systematic monitoring of the care process was reported as a facilitator in keeping track of the care progress. However, professionals described that in practice systematic monitoring was rarely conducted. They emphasized the need of concrete and usable monitoring instruments that facilitate professionals in structuring and keeping track of the care process.

### Evaluation with families

A reported facilitator was evaluation of the care process with families. Professionals described evaluation as improving families’ insight in the care process and positively influencing shared decision making on the type and intensity of support. Also, evaluation with families enabled professionals to keeping track of families’ changing needs and timely altering support if needed.

### Multidisciplinary case discussions

Weekly multidisciplinary case discussions within a Youth Team was a reported facilitator to evaluating care processes. According to professionals, multidisciplinary case discussions served multiple purposes: an objective approach of the care process and insight in potential blind spots; taking advantage of the broad expertise of the Youth Team; involving multiple perspectives in decision making; sharing responsibility with other professionals; and learning from each other. A barrier to multidisciplinary case discussions was the crisis-oriented focus of the cases discussed, leaving no room for other, less urgent, cases to be discussed. Subsequently, professionals described that this could lead to a lack of focus on scaling down and preventive activities, resulting in a risk of providing excessive support to families. Furthermore, a lack of structure during multidisciplinary case discussions was also stressed as a barrier, leading to inefficient meetings and dissatisfaction of professionals.

*“And that you regularly sit down with your colleagues and discuss ‘now I have done this, that has been achieved, and that does not work, and why does it not work? And what is the reason for trying again, if it has already been done?’ In this way, you stay sharp, I think that has added value.”*

- Professional HR1.4 –

### Evaluation of collaboration with other professionals

Frequent evaluation of collaboration with professionals of other organizations was described as a facilitator to integrated care. According to professionals, frequent evaluation resulted in improved agreements on roles, tasks, and working procedures, such as referral and care coordination.

## Discussion

To meet the needs of high-vulnerable families with severe and enduring problems across several life domains, professionals must improve their ability to provide integrated care timely and adequately. Based on the analysis of interviews with 24 professionals from multidisciplinary care teams in the Netherlands, we formed six themes covering facilitators and barriers these professionals encounter when providing integrated care (Appendix 2: Table 3). In general, there was consensus among professionals regarding the facilitators and barriers influencing their daily practice. Hence, the interviews were largely complementary and led to an in-depth thematic description of facilitators and barriers.

To tailor support to the changing needs of high-vulnerable families, professionals in our study stressed the importance of flexible and variable provision of support throughout the continuum of care by timely stepping up and scaling down. In line with previous research, multidisciplinary teams with a broad range of expertise and continuous pathways throughout the continuum of care were reported as facilitators to provide integrated care across several life domains [11, 12, 16]. The variety of barriers reported in this study highlight the complexity of supporting high-vulnerable families with chronic, unpredictable, and interacting problems across several life domains. As also found in previous studies, difficulties in prioritizing problems, allocating adequate support responsive to the changing needs of families, difficulties in interprofessional collaboration, and a lack of coordination over the care process hinders professionals to providing integrated care [8, 9, 13, 23].

Based on the thematic description of facilitators and barriers, we formulated five recommendations with implications for professionals, their organizations, researchers, and governmental policy makers that we believe are needed to address to further improve professionals’ ability to provide integrated care.

### Recommendation 1: Enhance knowledge of (potential) risks and needs of high-vulnerable families, to tailor care to family’s needs and identify gaps in the availability of support

As we conclude from the theme ‘Early identification and broad assessment’ and the theme ‘Current approaches in integrated care provision’, timely recognition of risks and needs is essential in providing integrated care. Enhancing our knowledge of potential risks and needs can improve insight in the type of expertise and support that is needed to cover families’ broad range of problems across several life domains. Furthermore, with this information, gaps in availability of support through the continuum of care can be identified. Echoing prior recommendations, availability of services throughout the entire continuum of care seems crucial to provide adequate, flexible, and enduring support for these high-vulnerable families [8, 11]. The lack of availability described in the themes ‘Continuous pathways’, ‘Multidisciplinary expertise’, and ‘Current approaches in integrated care provision’ is currently a major problem for professionals, since it forces them to provide support outside their scope of expertise. Formal agreements on tasks, roles, and responsibilities of professionals and their organizations during transition periods are needed to avoid overburdening of professionals when adequate support for families is unavailable.

### Recommendation 2: Increase professionals’ ability to broadly assess (potential) risks and address families’ needs, by being aware of their responsibilities as professionals and to timely involve others if needed

In addition to enhancing our knowledge of (potential) risks and needs, there is a need to increase professionals’ ability to broadly assess these risks and timely address families’ needs. Professionals in our study stressed that integrated care does not mean that one professional is responsible for *solving* all problems a family encounters. They described the importance of being aware of their professional responsibility to *identify* families’ potential risks and needs by early identification, broad assessment, and timely involve other professionals if needed. As can be concluded from the themes ‘Early identification and broad assessment’ and ‘Multidisciplinary expertise’, professionals need generalist expertise of a broad spectrum of problems, family dynamics, and potential risk factors. Hence, multidisciplinary teams seem to be an important facilitator to integrated care, since the diversity of all specialist expertise within a team leads to a broad range of generalist expertise. Moreover, professionals must be familiar with the broad variety of services in the field of Youth Care. However, it seems unrealistic that one individual professional can be familiar with all services throughout the continuum of care. Hence, to support professionals we strongly recommend organizations and policy makers to provide an up to date overview of available services on a local level.

### Recommendation 3: Keep professionals’ specialist expertise up to date and recognize the boundaries of their own expertise

Professionals in our study reported that they must keep their specialist expertise up to date to avoid a multidisciplinary team full of generalists. In that, organizations should facilitate the development and preservation of specialist expertise, for example by offering training and supervision. Furthermore, as described in the theme ‘Multidisciplinary expertise’, professionals should be aware of the reach and boundaries of their specialist expertise to preserve high quality integrated care. Multidisciplinary case discussions were reported as facilitators to increase insight in potential blind spots and learn from the broad expertise represented within the Youth Team. However, previous research on learning activities reported that training, supervision, interprofessional learning, and frequent evaluations were hindered by difficulties in prioritizing, high work demands, or a lack of time [38]. Therefore, professionals and organizations should collaboratively discuss options for effectively executing these learning activities, for example by scheduling monthly evaluative meetings.

### Recommendation 4: Facilitate professionals in timely stepping up and scaling down by improving systematic monitoring and frequent evaluation of care processes

As can be concluded from the theme ‘Current approaches in integrated care provision’, professionals seem to offer a mix of matched and stepped care when providing integrated care. They tailor support to families’ needs and preferences, while starting with the least restrictive support as possible, and gradually increase the intensity of support if needed. Professionals reported timely stepping up to more intensive support and scaling down to less restrictive support as a necessity to provide integrated care. Interestingly, professionals often attributed difficulties with stepping up to external factors such as a lack of availability of support, whereas difficulties with scaling down were attributed to internal factors such as professionals feeling responsible, personal involvement, and the concerns regarding the risk of relapse in high-vulnerable families. Hence, to overcome difficulties in stepping up and scaling down, it is important for professionals to recognize and distinguish these internal and external aspects. In line with previous research, frequent evaluation of the care process was reported as a facilitator to adequately decide on the focus of support and timely alter support if needed by stepping up or scaling down [16, 18]. However, professionals in our study mentioned that the care process was rarely monitored in practice and evaluations often lacked structure. Furthermore, the crisis-oriented focus during multidisciplinary case discussions led to a lack of focus on scaling down and preventive activities. This is especially critical in supporting high-vulnerable families, since the chronic, unpredictable nature of interacting problems and reoccurring crisis situations requires systematic monitoring and frequent evaluation [3]. Besides sufficient resources for evaluation such as time and monitoring instruments, future practice-based studies should focus on identifying facilitators and barriers that professionals encounter during multidisciplinary case discussions to guide professionals in improving these evaluations.

### Recommendation 5: Find balance between the use of guidelines and a professional’s autonomy to tailor support to families’ needs

Lastly, as described in the theme ‘Autonomy of professionals’, a professional’s autonomy to undertake a variety of tasks is a facilitator to tailor support to families’ needs. However, many professionals were concerned that too much autonomy led to intuitive decision making and varying working approaches, resulting in inadmissible variations in the support of families with similar problems. A remarkable finding was that few professionals mentioned the use of (evidence-based) guidelines in their daily practice, since guidelines can provide structure, focus, and equality in care processes [13]. What professionals did report was that strict guidelines on the duration of support and the number of visits was a barrier to tailor support to families’ needs. As we already know from previous research, structured protocols and guidelines for example used in stepped care, do not always match the pace of families and overlook the interaction of problems that high-vulnerable families encounter [23, 25]. Therefore, we advocate that there is a need to collaboratively improve practice-based and evidence-based guidelines concerning the content of support for high-vulnerable families. For example, these guidelines can support professionals in prioritizing problems, allocating adequate support responsive to the changing needs of families. Importantly, these guidelines should assist professionals in structuring the care process and working effectively by a goal-oriented approach, while similarly leaving a certain degree of freedom and flexibility to tailor support to the needs of families.

### Strengths and limitations

An important strength of this study lies in the fact that qualitative research provides a powerful methodology for exploring complex processes and thereby facilitates a deep understanding of professionals’ perspectives on integrated care [33]. In total, we interviewed 24 professionals from Youth Teams in The Netherlands. Although professionals were predominantly female, this male–female ratio reflects the usual sex proportions in Youth Teams. The interviews provided complementary information, resulting in a rich description of facilitators and barriers professionals encounter when providing integrated care. By applying the COREQ guidelines [32], we ensured systematic and transparent reporting of our study methods and interpretation of the results. The structured analysis procedure, guided by a theoretic framework and open coding, enhanced the comprehensiveness of the results. Also, the iterative process of analysis, the use of subjective expressions of participants (quotes), and the reflexive meetings enabled us to explore the data in depth and decreased the risk of researchers’ subjectivism [35].

On the other hand, several limitations must be considered. The most important limitation lies in the fact that the interviews were conducted during a restrictive period in a highly changing context. Together with the narrow focus on a group of professionals working in Youth Teams in the Netherlands, this decreases the transferability of the results and complicates the assessment of data rigidly. Therefore, it will be interesting to repeat the interviews at another time or within another population of Youth Care professionals. Moreover, to further our understanding of the extent to which these facilitators and barriers influence clinical practice, there is a need for high-quality mixed-methods research.

## Conclusions

Taken together, this qualitative study highlights the need for flexible support across several life domains to meet the needs of high-vulnerable families. To substantially improve professionals’ ability to support these families, we formulated five recommendations based on the facilitators and barriers professionals encounter when providing integrated care. First, research should enhance our knowledge of (potential) risks and needs. Then, organizations and professionals should invest in improving professionals’ ability to broadly assess these (potential) risks and needs of high-vulnerable families. Also, professionals’ specialist expertise should be kept up to date to avoid a multidisciplinary team of generalists. Moreover, to facilitate professionals in timely stepping up and scaling down, systematic monitoring and the evaluation of care processes should be improved in practice. Finally, practice, research, and governmental policy should find a balance between the use of guidelines to structure a care process and a professional’s autonomy to tailor support to families’ needs.

## Data Availability

The datasets used and analyzed during the current study (transcript of the interviews) are not publicly available due to privacy related issues. Information on transcripts is available from the corresponding author on reasonable request.

## Appendices

### Appendix 1

See Table 2.

**Table 2:** Coding framework

Concept	Category	Code	Frequency of quotes per code	Description	Literature
Integrated care	General principles	Coordinated	40	Coordination in the care process across professional, organizational, and system boundaries	World Health Organization [4]
		Coherent	11	Coherence in assessment and support, across professionals and in policies	World Health Organization [4]
		Continuity	18	Continuous support over time (within and between professionals)	World Health Organization [4]
		Family focused	42	Addressing the needs of all family members	Tausendfreund et al. [3]
		(Lack of focus on) several life domains	33	(Lack of) focus on several life domains: academic, familial, social and personal	Tausendfreund et al. [3], Wang et al. [27]
		Interprofessional collaboration (intern or extern)	79 (extern) 46 (intern)	Collaboration between professionals involved in the care process Intern: collaboration with professionals within the own care team. Extern: collaboration with professionals from other organizations	Cooper [8], Hermens et al. [12], Janssens et al. [11], Van Straten et al. [13]
	Expertise	Generalist/Specialist expertise	50	Broad knowledge and approach of problems (generalist) or in-depth knowledge and approach of problems (specialist)	Hoffses et al. [9]
	Assessment	Early identification/Early assessment	14	Timely recognition of (potential) risk factors across several life domains	Bower and Gilbody [15], Linton et al. [14], Van Straten et al. [13]
		Broad assessment	36	Assessment of a broad range of problems across multiple life domains	Bower and Gilbody [15]; Linton et al. [14]; Van Straten et al. [13]
		Multiple, co-occurring problems	26	Interaction between multiple problems that occur simultaneously	Henderson et al. [23]; Tausendfreund et al. [3]
	Service delivery	Availability of support	78	Availability of support throughout the continuum of care	Cooper et al. [8]; Meeuwissen [16]
		Continuous clinical pathways/Fragmented care	48	Clear, non-fragmented routes of care through the entire continuum of care (universal services to primary care to specialized secondary care)/fragmentation between services or professionals	Cooper et al. [8], Hermens et al. [12]; Meeuwissen et al. [16]
Stepped care	Definition	Stepped care (definition)	4	Offering the least restrictive support as possible that is still likely to yield significant health gain and step up to more severe care if necessary	Bower and Gilbody [15], Meeuwissen [16], Bennett-Levy et al. [17]
	Allocation of interventions	Predetermined sequence	7	Support ranked from low to high intensity in a predetermined sequence	Firth et al. [18], Meeuwissen [16], Richards [19]
		Least restrictive	18	The least intensive support in terms of time, costs, and professional’s level of expertise	Meeuwissen [16], Van Straten et al. [13]
		Intensity	14	Providing support by a predefined sequence of support options with increasing intensity	Bower and Gilbody [15], Firth et al. [18], Meeuwissen [16], van Straten et al. [13]
	Assessment and evaluation	Reflexive monitoring/(ir)regular monitoring	15	Progress and outcomes are monitored by collecting data to assess if support must be altered	Meeuwissen [16], Richards [19], Bower and Gilbody [15]
		(standardized and systematic) Evaluation	42	Periodically and systematically evaluate progress in a care process and collaboration	Van Straten et al. [13], Meeuwissen [16], Firth et al. [18], Bower and Gilbody [15]
		Goal efficiency	14	Working efficiently towards concrete goals	Meeuwissen [16]
	Disadvantage stepped care	Focus on individuals/single problems	3	Focus on individuals and single problems, omitting the complex interaction of problems	Cross and Hickie [25]
		Variety in steps	5	Stepped care support is heterogeneous with different numbers of steps, intensity, and treatment components	Van Straten et al. [13], Bower and Gilbody [15]
		Lack of predefined criteria/guidelines	41	Lack of predefined criteria and (clinical, practical, or evidence-based) guidelines for monitoring and evaluation of support hinder stepped care	Meeuwissen [16]; Van Straten et al. [13]
		Under treatment	33	Inappropriate support or inefficient allocation of resources leading to an exacerbation of family’s problems	Linton et al. [14], Lovell and Richards [22]
		Risk of drop out	10	Families refusing further support	Seekles et al. [26]
Matched care	Definition	Matched care (definition)	16	Allocation of support is based (matched) on families’ characteristics, preferences, risks, and needs	Van Straten et al. [13], Linton et al. [14]
	Allocation of interventions	Tailored	52	Family’s needs and preferences are central in the allocation of support	Van Straten et al. [13], Linton et al. [14]
	Disadvantage matched care	Lack of prognostic determinants	2	Lack of clear prognostic determinants to match families to the available support	Bower and Gilbody [15]; Van Straten et al. [13]
		Variety of interventions	18	Support may vary across families regarding intensity, setting, and type of professional	Linton et al. [14]; Van Straten et al. [13]
		Overtreatment	13	Families receiving too many support, leading to inappropriate allocation of services	Lovell and Richards [22]
Decision making	Decision making	Shared decision making	27	Shared decision making is based on collaboration between professionals and families, taking families’ preferences into account and jointly decide the type and intensity of support	Meeuwissen [16]; Van Straten et al. [13]
		Intuitive decision making	27	Intuitive decision making, not based on reflexive monitoring, evaluation, or predefined determinants	Meeuwissen [16], Van Straten et al. [13]
Quality of services	Service delivery	User friendliness	10	Satisfaction with- and user friendliness of support	World Health Organization [4]
		Safety	26	Professionals paying attention to a family’s safety	World Health Organization [4]
Open coding		Freedom of professional	28	A professional’s freedom to make her/his own decisions in the care process	
		Solution focused approach/therapy	16	Support that focuses on solutions rather than problems	
		Familiarity	50	Familiarity with other services or professionals (often affects the feeling of availability)	
		Trust	30	Trust between professionals	
		Early consultation	37	Early consultation function of professionals in for example schools to provide early support	
		Care plan	18	Care plan with goals for the entire family	
		Clinical case discussion	35	Clinical case discussions within multidisciplinary care teams to discuss and evaluate the care process	
		Stepping up	52	Step up to more intensive support if needed	
		Scale down	46	The opposite of stepping up, the provision of less restrictive support after intensive support	
		Integrated care definition/in general	29	Definition of integrated care, general aspects of integrated care	
		Warm handoff	14	The gradual transfer from one professional to another	

### Appendix 2

See Table 3.

**Table 3:** Overview of facilitators and barriers per theme

	Theme	Facilitators	Barriers
1	Early identification and broad assessment to timely recognize potential risk factors	Early consultation Awareness of (potential) risk factors Accessibility and availability Addressing broad range of topics in broad assessment Outreaching approach Shared care plan	Risk of providing excessive support for minor problems Lack of knowledge of a broad range of problems Time consuming and burdensome for families
2	Multidisciplinary expertise: specialist professionals in a generalist team	Awareness of the reach of a professional’s own expertise Multidisciplinary teams: work in pairs Keeping specialist expertise up to date	High working demands, forcing professionals to provide support on areas outside their expertise
3	Continuous pathways: flexible support throughout the entire continuum of care	Familiarity with other professionals by co-location and joint case discussion Frequent evaluation and agreements Sharing up to date information Warm handoff between professionals A care coordinator	Complexity and variability of problems Unclear tasks, roles and responsibilities Time consuming Specific organizational demands Privacy issues in sharing information Lack of availability of professionals and high turnover rates Lack of availability of support due to long waiting lists Limited availability of support for specific ethnic groups
4	Current approaches in integrated care provision: a mix of stepped and matched care	Providing different options for support Tailor care to families’ needs and preferences Shared decision making Guide families through decision making process Future oriented (shared) care plan Early involvement of the informal network and schools Frequent evaluation of a family’s progress	Least restrictive support inappropriate Time-limit for each step, not matching the pace of families and hence support is not tailored to their needs Difficulties early assessment Lack of availability of support Resistance of families towards less restrictive support of scaling up Limited attention to scaling down Difficulties in objective assessment during crisis-situations Sense of responsibility and personal involvement
5	Autonomy of professionals: tailor support and follow guidelines	Autonomy to undertake a variety of tasks to tailor support Focus on professionals’ competencies and personal development Discussing focus of support in multidisciplinary team Structure and extended expertise by following guidelines	Too much autonomy leads to unclear tasks, responsibilities and insecurity Inadmissible differences between professionals in type of support provided Fixed protocols limits the autonomy of professionals Intuitive decision making
6	Evaluation of care processes: discuss progress and alter support if needed	Systematic monitoring of the care process Concrete, usable monitoring instruments Weekly clinical case discussions Evaluation of collaboration with other professionals Evaluation of the care process with families	Lack of systematic monitoring Crisis-oriented focus in case discussions Lack of focus scaling down and preventive activities Lack of structure during clinical case discussions
